# Post-Pyrolytic Carbon as a Phase Change Materials (PCMs) Carrier for Application in Building Materials

**DOI:** 10.3390/ma13061268

**Published:** 2020-03-11

**Authors:** Michał Ryms, Katarzyna Januszewicz, Paweł Kazimierski, Justyna Łuczak, Ewa Klugmann-Radziemska, Witold M. Lewandowski

**Affiliations:** 1 Department of Energy Conversion and Storage, Faculty of Chemistry, Gdansk University of Technology, G.Narutowicza 11/12, PL-80-233 Gdańsk, Poland; 2 Institute of Fluid Flow Machinery, Polish Academy of Sciences, Fiszera 14, PL 80-231 Gdańsk, Poland; 3 Department of Process Engineering and Chemical Technology, Faculty of Chemistry, Gdansk University of Technology, G.Narutowicza 11/12, PL-80-233 Gdańsk, Poland

**Keywords:** PCM, phase-change materials, activated carbon, PCM carriers, biomass pyrolysis, thermal stability, building materials modification

## Abstract

This article covers new application for char as a carrier of phase-change materials (PCM) that could be used as an additive to building materials. Being composed of bio-char and PCM, the granulate successfully competes with more expensive commercial materials of this type, such as Micronal^®^ PCM. As a PCM carrier, char that was obtained from the pyrolysis of chestnut fruit (Aesculus hippocastanum) with different absorbances of the model phase-change material, Rubitherm RT22, was tested. DSC analysis elucidated several thermal properties (such as enthalpy, phase transition temperature, and temperature peak) of those mixtures and the results were compared with a commercial equivalent, Micronal DS 5040 X. Comparative research, approximating realistic conditions, were also performed by cooling and heating samples in a form of coatings that were made from chars with different content of RT22. These results indicated that the use of char as a PCM carrier was not only possible, but also beneficial from a thermodynamic point of view and it could serve as an alternative to commercial products. In this case, adsorption RT22 into char allowed for temperature stabilization comparable to Micronal DS 5040 X with ease of use as well as the economic advantages of being very low cost to produce due to microencapsulation. Other advantage of the proposed solution is related with the application of char obtained from waste biomass pyrolysis as a PCM carrier, and using this product in building construction to improve thermal comfort and increase energy efficiency.

## 1. Introduction

Analyzing the development directions of modern building construction, there is a clear effort to build with attention towards a reduction in energy consumption without sacrificing comfort [1,2,3]. We increasingly seek to develop new materials with better thermal and insulation properties that could be used in building construction because we attach so much importance to reduce energy consumption, optimizing its use and searching for new, pro-ecological ways to obtain it. Current building materials do result in significant energy consumption reduction; however, they are still relatively expensive and require new solutions not only to reduce energy consumption, but also to be more cost effective [4,5,6,7]. As conventional building material selection occurs primarily with cost in mind, one such example comes from buildings in Central and Eastern Europe, which are primarily made from silicate blocks with moderate thermodynamic properties. Styrofoam is also a popular supportive building material that works with silicate blocks and also lowers energy consumption, but, due to the possibility of thermal bridges between the contacting blocks, lowers energy consumption to a certain level. In low-energy, and especially passive building constructions, materials with better insulation properties should be used, but those materials are more expensive and drive up investment/construction costs. In addition to improving the insulation of materials used in energy-efficient buildings, they can be modified by addition of phase-change materials (PCM), which further improves the energy balance of that building [8]. Unfortunately, despite commercial availability, these solutions have not been widely adopted, due to their high price. Therefore, it is necessary to look for opportunities to develop inexpensive building materials, which can not only prevent energy loss in the first place, but temporarily store excess energy (like solar energy) for release in a timely manner. Such solutions have already been analyzed many times [9,10,11,12,13,14]; with several proposed solutions [15,16,17,18]. However, ongoing research in this area is still critical and valued in the building construction industry.

There are many known methods for the practical use of phase-change materials, especially in building construction, where microcapsules are most commonly used [19,20,21,22,23,24,25,26,27] (e.g., Micronal 5040X, i.e., Rubitherm RT22 trapped inside a polyethylene coating). These microcapsules, mixed with gypsum, cement, or mortar, are included in gypsum walls, mortars, screeds, or plasters. The disadvantages that are associated with these materials include: high price (due to difficulty obtaining the Micronal product), low thermal conductivity, and low mechanical strength. At higher loads, those problems result in cracking and the depressurization of polymer coatings and pouring of the PCM after exceeding its melting point.

This last drawback does not appear in the solution using metal, ceramic, glass, or other hermetic containers filled with PCM, the mechanical strength of which can be adjusted by wall thickness. However, they have a high thermal inertia, due to the low thermal conductivity of materials that are solidified within. This limits their application in heat storages and regenerators, in which fast-changing thermal energy occurs. Additionally, in building construction, due to the need to redesign traditional building material shapes (bricks, hollow bricks, etc.) to utilize PCM materials within them has hampered the development of this approach.

There are some known attempts of using lightweight aggregate (LWA), e.g., Pollytag^®^ as a carrier [28,29] with PCM (Ceresin) completed with a patent [30]. PCM that was trapped in the LWA pores was able to stabilize the temperature of the asphalt road surface in laboratory conditions at a lower level, when compared to a surface without PCM. This behavior could theoretically protect the asphalt mineral mixture from softening and rutting under real conditions. However, this solution has not yet been put into practical use due to implementation difficulties related to the mechanical strength of the resulting products. A relatively low amount of Ceresin (3% by weight) adsorbed into the pores of Pollytag^®^ construction aggregate; however, achieving the required stabilization temperature necessitated the addition of large amounts of modified aggregate into the asphalt mixture, which lowered its mechanical strength when compared to the traditional mineral aggregate and reduced the pavement surface hardness. Research on the use of LWA and PCM was also conducted relative to traditional building materials [14], but, as a carrier, its use has encountered some limitations due to the specificity of Pollytag^®^. First, the air remaining in its pores hindered heat exchange and inhibited PCM phase changes. The second limitation was the relatively large granulate size, which poses an obstacle when adding it or filling traditional building materials with it (holes, hollow bricks, etc.). However, filling with this granulate gypsum wall or plaster, as well as covering the surface with it, was already problematic.

In this paper, the use of post-pyrolytic carbonate that was obtained from waste biomass as a PCM carrier is proposed. The effects of the above solution were verified in an experimental way, examining the heating and cooling time of the coatings that were applied to the temperature sensors made of char containing various amounts of Rubitherm (PCM), wherein one of the coatings, made from the char without PCM, was taken as a reference sample. Although the experiments presented in this manuscript were mainly limited to coatings, the material can also be implicitly used in a wide range in construction.

## 2. Theoretical Background

The starting point for these considerations were studies on the possibility of heat accumulation using phase change materials that were previously mentioned, as it related to modified asphalt pavement for its temperature stabilization [28,29]. Lightweight aggregate was then used as the PCM carrier and it provided a porosity that was high enough to absorb a sufficient amount of PCM with acceptable strength. This solution has patent protection [30] and it paves the way for other similar applications such as classic building construction, in which solutions based on the PCM sealing modification in containers, capsules, bags, or microgranules as a protection against potential leakage had been most common. In these solutions, PCM was trapped in porous aggregate and, thanks to the van der Waals forces, remained in LWA, even at high temperatures and it was experimentally confirmed [14]. Those experiments showed that use of PCM mixtures with an aggregate additive had a positive effect on temperature stabilization of other building materials and improved the thermal comfort of rooms. For hollow blocks and gypsum blocks that were modified with the aggregate and PCM mixture, a significant extension of the temperature stabilization period (thermal comfort) was achieved, but depended on the initial conditions [14]. Aside from cost reduction, this modification additionally resulted in resource savings through the replacement of expensive yet easy to use Micronal DS 5040 X with a less expensive and more durable alternative—Rubitherm RT22 adsorbed on a cheap and mechanically durable carrier, which would be difficult to apply directly in liquid form. This new solution enables the easy implementation of phase-change materials in construction.

A natural consequence of research on PCM carriers is the search for novel materials with high porosity and environmental friendliness. One of those novel materials might be char, which, through activation and increased absorption capacity, has potential as an excellent PCM carrier. Another advantage is its production via pyrolytic utilization as both waste biomass and municipal waste combustion (RDF; tires, sewage sludge, etc.). The possibility of valorization and use of waste products that are currently incinerated is a commercially attractive research direction.

The purpose of the research described is the use of char obtained after pyrolysis as a PCM carrier. It was assumed the simplest solution would involve adsorption that was carried out above the PCM melting point. The same char was used in this study; however, the resulting product was comprehensively examined for its physical and thermodynamic properties as well as its potential in practical application by comparing synthesized materials with commercially available microcapsules containing the same PCM due to different activation methods showing a different specific surface areas and different absorption capacities. Heating and cooling this modified char allowed for a determination of the hysteresis and repeatability of the process as well as its durability, which certainly impact potential applications, such as building, road construction, and even gardening.

The amount of PCM, relative to the total weight of building materials, determines the effectiveness of temperature stabilization within a building. For example, in tests that were carried out with a 3% (by weight) addition of ceresin trapped in LWA [29], the decrease in surface temperature, as compared to parallel measurements for surfaces without ceresin, was ~5 K. In this work, in which the PCM share the biocarbonate, should be 5–7 times higher, which represents an adequate increase in the thermodynamic effect or a reduction in the amount of modified PCM carbonate added to building materials (gypsum, cement, mortar, etc.).

In this study, a product of waste biomass pyrolytic decomposition was used; in this case, it was the fruit of inedible chestnut (*Aesculus hippocastanum*), although the general source of the char might originate from any type of waste biomass, which increases the rationale for undertaking research in this area. It is worth emphasizing that the formation of the adsorptive structure of the char begins during the thermal, anaerobic biomass degradation. Parameters, such as temperature, presence of inert gas, heating rate, and reactor type, have a significant impact on the quantity and quality of pyrolysis products [31,32]. The wide range of available pyrolyzers for both laboratory and technical scale applications allows for their selection in terms of the desired product properties.

A muffle furnace was used due to the laboratory scale of this work. It was assumed that the char that was obtained after these processes might not have sufficiently developed a surface, which negatively impacted its adsorption properties. Therefore, several activation pathways were examined to improve the carbonate sorption parameters. These pathways unblocked their pores, being often blocked by gaseous and liquid post-pyrolytic residues, which resulted in porosity and specific surface increases. Char, like any activated carbon, has a specific surface area, depending on its porosity (macro-meso and micropores), which can be increased by activation. The greater the ratio of the total area of all pores to the mass of activated carbon, the more extensive its structure and the greater its absorption potential.

Activation is divided into two classifications—physical, where the activator can be carbon dioxide, steam, and/or oxygen at temperatures from 800–1000 °C; and, chemical, where the activator is sodium hydroxide, zinc chloride, and phosphoric acid (V) at temperatures from 400–1000 °C. An advantage of chemical activation is that is a one step process in conjunction with biomass pyrolysis previously impregnated with the activator. In the tests described below, the effect of the surface area development and pore size on PCM adsorption was assessed; chemically and physically activated samples were used.

It is expected that the insertion of PCM in place of air into porous char will increase its mechanical strength, especially pressure and hardness; where strength, depending on the type of PCM, might even surpass that of the biomass itself, e.g., wood from which this char can be obtained. These features, in addition to better thermal conductivity, should also provide a definite advantage for the char as a PCM carrier, relative to other carriers (Pollytag^®^, Leca^®^ KERAMZYT, aerated concrete, porous gypsum to name a few), and over encapsulated PCM, foil, or placed in containers. 

## 3. Methodology and Experimental Study

The methodology in this work is difficult to formally describe without reference to individual experiments, as it was different for each test. Utilized methodologies include:

- pyrolysis of various types of waste biomass (N_2_—inert gas, temperature: 800 °C, time: 90 min.—Section 3.1);
- determining the composition, as well as physical and sorption properties of the obtained biochar (proximate and elementary analysis);
- methods used to activate the biochar (Section 3.2) and study the effects of increasing the sorption surface (BET surface and pore size analysis);
- PCM sorption procedure in biochar (typical vacuum sorption in liquid Rubitherm RT22 for approx. 30 min., and thermal draining at 80 °C in the same time period);
- testing and selecting biomass in terms of the sorption capacity of the biochar that was obtained from it, from currently available biomass types: coconut shell, walnut shell, chestnuts (validation of the sorption capacity of various types of activated and inactivated biochars, based on DSC analysis—Section 3.3); and,
- conducting experimental studies of thermodynamic properties of a material obtained from selected biochar (chestnuts), modified by PCM (Section 3.4).

In this situation, while considering the accessibility and readability of the work, the authors decided to provide information on the methodology successively, within the individual descriptions of the experiments.

### 3.1. Preparation of Post-Pyrolytic Carbon

Biomass is required to obtain char as a PCM carrier, which can be obtained from various sources. From ecological and economical points of view, this should be waste biomass that is intended for disposal.

Therefore, chestnut fruits (*Aesculus hippocastanum*), often referred to simply as chestnuts—an inedible resource often subjected in Poland to waste disposal—was selected as the biomass example for this study.

The process of obtaining char was as follows. Chestnuts were dried for 12 h at 105 °C and ground using a knife mill (3 mm mesh diameter). Pyrolysis of ~100 g of the granulate was carried out without an inert gas flow for 90 min. at 800 °C in a steel reactor with an outlet tube placed in a muffle furnace. The solid recovery after pyrolysis was 25% of the initial mass.

After mixing several batches, the elemental analysis of chestnut fruit and the char obtained from it was determined (Thermo Scientific™ FLASH 2000 CHNS/O Analyzer, Thermo Fisher Scientific Inc., Waltham, MA, USA) as well as basic property tests (Table 1). Elemental analysis showed an increase in elemental carbon (relative to the original biomass), while the hydrogen, oxygen, and sulfur levels all decreased, which indicated the release of volatile compounds. Precursors with a high levels of elemental carbon enable effective activation.

Table 1 presents the proximate (moisture, ash, volatiles) and elemental analysis (CHNSO) of the raw material (chestnut) used for the pyrolysis experiments. The moisture content was about 3.5 wt.% (the sample was dried before analysis). The volatile matter was detected to be 74.6 wt.%, whereas the ash content was 3.5 wt.%. For comparison purposes, in the same table (Table 1), the results that were obtained for the solid product of the pyrolysis (char) were also included. The thermal degradation of raw material removed the volatile compounds from the sample (CO_x_, H_2_O, H_2_), which resulted in lower hydrogen and oxygen content.

### 3.2. Char Activation and Surface Analysis

The carbonate was chemically activated using KOH and physically with carbon dioxide to improve sorption parameters. Chemical activation consisted of mixing the char with KOH in a 3:1 weight ratio, followed by heating in a tubular furnace at 800 °C for approximately 90 min.; this mixture was comminuted in a mortar. Unlike pyrolysis, the activation process took place under a stream of nitrogen. The mass ratio of the activating agent to char was chosen based on previous results from our own work. After adding 50 mL of deionized water, the sample was purified in an ultrasonic bath (30 min.) from excess KOH, washed with 5 M HCl and deionized water until pH 7, and then dried for 24 h at 105 °C.

The activation mechanism of KOH char is described, depending on the temperature, by the following primary reactions [33]:

2 KOH (below 700 K) → K_2_O + H_2_O,

C + H_2_O → CO + H_2_

CO + H_2_O → CO_2_ + H_2_

CO_2_ + K_2_O → K_2_CO_3_,

6 KOH + 2 C (under 900 K) → 2K + 3H_2_ + 2K_2_CO_3_,

which may also be accompanied by secondary reactions:

C + CO_2_ → CO

C + K_2_O → 2K + CO

2C + K_2_CO_3_ → 2K + 3CO.

Physical activation of ~20 g samples were carried out in a tubular furnace at 800 °C under a stream of carbon dioxide (10 dm^3^/h). Two physical activation series of CO_2_ carbonized char were performed; the difference between them was the washing times with the activating agent (1 h, 10 dm^3^ of CO_2_; 2 h, and 20 dm^3^ of CO_2_). The physical activation mechanism is described by an endothermic (ΔHº_298_ = +172.42 kJ/mol) Boudouard reaction:

C + CO_2_ = 2CO.

The simultaneous assessments of sorption properties and the effectiveness of the activation process were performed using surface and pore size analysis; the Brunauer-Emmett-Teller (BET) was determined by N_2_ absorption-desorption isotherms at 77 K while using a Micromeritics Gemini V200 Shimadzu analyzer (Table 2).

The biochar samples were activated using two main methods, namely chemical and physical activation (10 and 20 dm^3^ of CO_2_ used in 1 h and 2 h of activation process). The amount of activating agent was calculated proportionally to the carbon content in the sample (taken from the elemental analysis, Table 1). In this regard, CO_2_-to-carbon molar ratio was set to be 1:0.5 and 1:1. The efficiency of activation was evaluated by the BET surface analysis (Table 2). As the reference sample, inactivated biochar with a surface area of 31.6 m^2^/g was used. It was found that the surface area and volume of pores of biochar after physical activation performed at 1000 °C using CO_2_ (sample III) increased with the amount of CO_2_ used in the experiment (sample IV). Nevertheless, the highest S_BET_ was obtained for the sample that was chemically activated with KOH. The surface area achieved was 1252.5 m^2^/g with the pore volume of 0.64 cm^3^/g.

The significantly improved performance was observed when KOH was applied while analyzing the effect of developing the porous structure due to the activation. However, the physical activation process was more economical, as the amount of activator and its cost (exhaust gas) is negligible.

### 3.3. Thermal Calorimetry Study

Another component of this research was a PCM introduction (commercially available Rubitherm RT22) to various samples of the carbonate: II—inactivated, III—activated 1 h with CO_2_, IV—activated 2 h with CO_2_, and V—activated with KOH. Char sample I, as a reference, was neither activated nor containing PCM. The synthesis began with ~2 g of the char was added to the molten Rubiterm RT22 and stirred for 1 h at 80 °C. The excess Rubiterm was vacuum filtered and the samples were dried on paper at 80 °C for 12 h. This procedure was utilized for all samples, except sample I. In this way, only PCM remained permanently adsorbed by the van der Waals forces in samples II–V.

**Figure 1:**
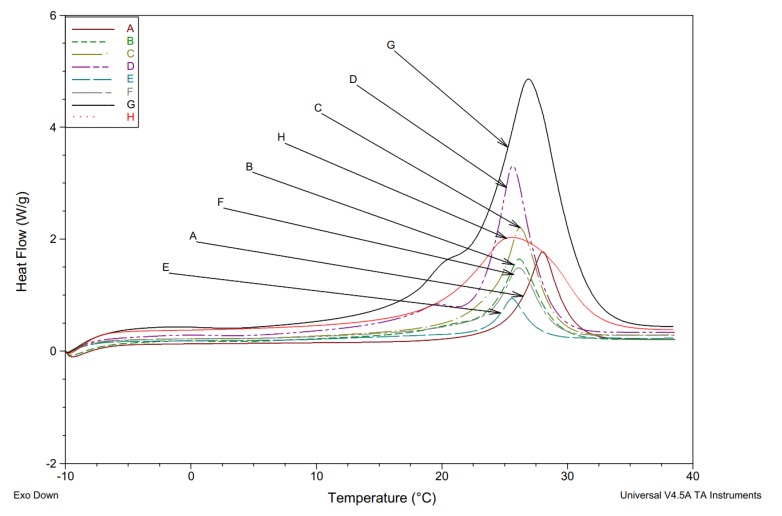
DSC diagram presenting results of testing char samples soaked with Rubitherm RT22 and reference samples, including; carbonate from chestnut fruit: A (sample II)—not activated, B (sample III))—activated with CO_2_ (1 h), C (sample IV) )—activated with CO_2_ (2 h), D (sample V) )—activated with KOH; char from: E)—coconut shell activated with CO_2_ (1/2 h), F)—walnut shell activated with CO_2_ (1/2 h); and, reference samples: G)—pure Rubitherm RT22 and H)—Micronal DS 5040 X.

Thermal comparison tests were performed using a DSC (Differential Scanning Calorimeter Q20, TA Instruments-Waters LLC, USA) from −90 to 450 °C with a temperature accuracy of ±0.1 °C, precision of ±0.01 °C, and enthalpy precision of ±0.1%. A preliminary comparison was carried out for six different carrier types of post-pyrolytic biomass carbonate (chestnut fruit, coconut, and walnut shells), without activation, or activated by four different methods. A 90 min. CO_2_ activation was added in addition to the methods of physical activation discussed above. Three different samples of the same substance were tested each time for DSC tests. These were subjected to six complete runs (three times cooling and three times heating) in the temperature range between −10 and 40 °C. Figure 1 shows the averaged results of those comparisons for heating cycle (exo down).

The comparative analysis of thermographs that is shown in Figure 1 indicated, as expected, that pure Rubitherm RT22 showed the best heat storage properties. The commercial product Micronal DS 5040 X was next, despite a drop in heat capacity of ~60%. However, results obtained for chestnut char that were subjected to KOH activation showed similar heat capacities (~50% relative to RT22) and an almost identical temperature per peak maximum of 25 °C. Table 3 summarizes a detailed summary of DSC test results for individual samples, the biomass origin, and how it was activated.

### 3.4. Thermodynamic Study 

The DSC results confirmed that, from a thermodynamic point of view, it is possible to use char as a PCM carrier and also indicated the most beneficial type of biomass based on the method to activate the char. However, it was decided to conduct tests on a laboratory scale to confirm these DSC results before executing expensive and time-consuming tests on a technical scale using real building materials.

**Figure 2:**
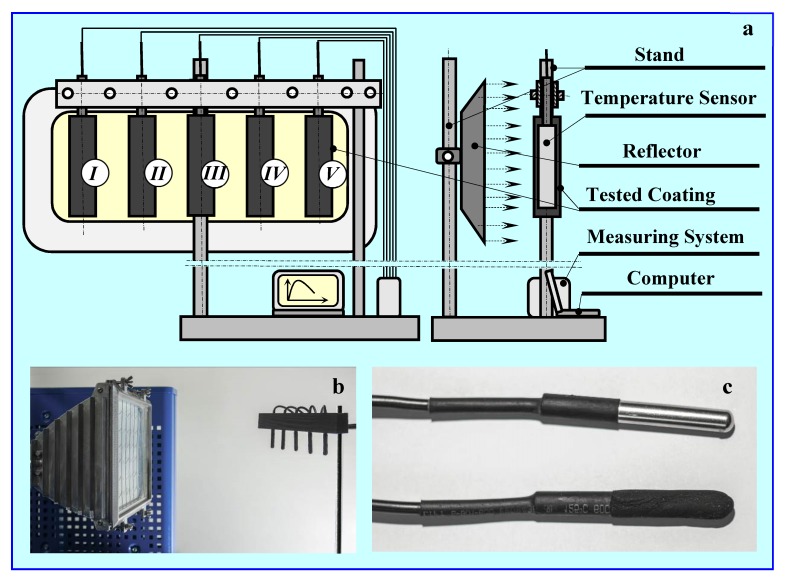
Measuring the heating and cooling times of the tested coatings with infrared radiation. (**a**) Scheme of measuring station, (**b**) view of the reflector and stand, (**c**) temperature sensors without and with coating. For cooling, after placing, exposing and heating the samples, no simulator was needed. Composition of individual char coatings: I—without RT22, II—with RT22, III—activated with CO_2_ (1 h), including RT22, IV—activated with CO_2_ (2 h), including RT22, V—activated with KOH, including RT22.

For this purpose, a measuring station (Figure 2) was built to study the heating and cooling times of coatings made using the most thermodynamically promising char that was obtained from pyrolysis of common nut fruit. This was, as shown by earlier DSC tests, the absorption of Rubitherm RT22 and depended on the activation type or lack thereof.

**Figure 3:**
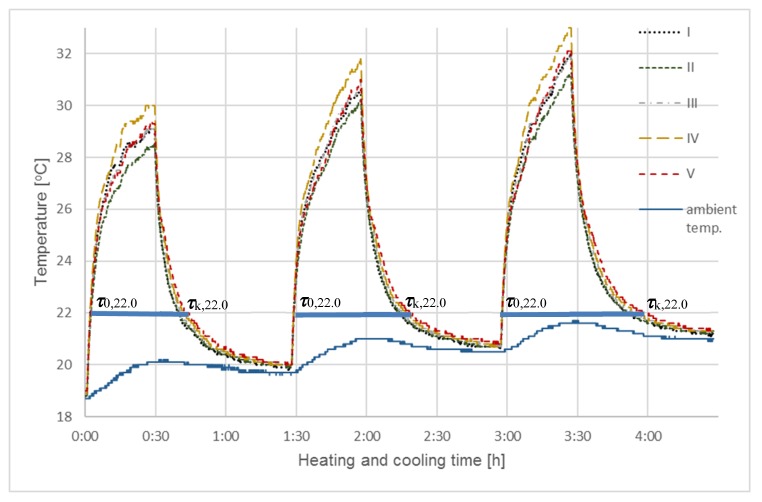
Testing of coatings using a solar lighting simulator - SI. The diagram shows results of the heating and cooling steps, for 0.5 h and 1 h, respectively, for five chestnut char samples: I—without activation and without RT22, II—without activation with RT22, III—activation with CO_2_ (1 h) with RT22, IV—activation with CO_2_ (2 h) with RT22, V—activation with KOH with RT22.

**Figure 4:**
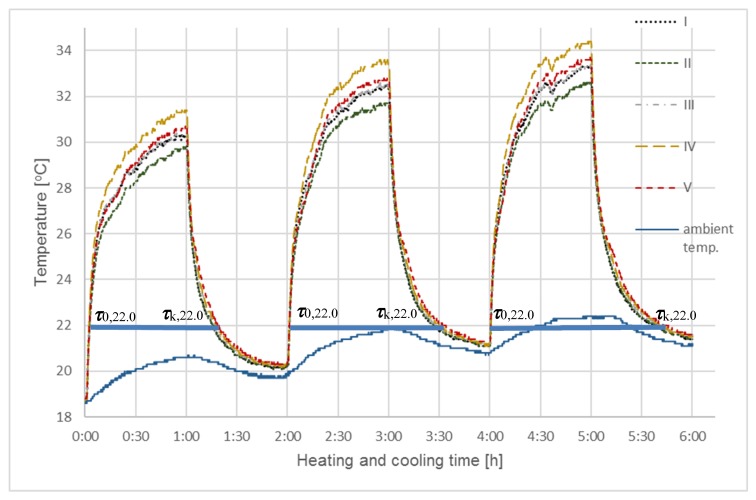
Testing of coatings using a solar lighting simulator - SI. The diagram shows the results of the heating and cooling process for each of five chestnut char samples for 1 h: I—without activation and without RT22, II—without activation with RT22, III—activation with CO_2_ (1 h) with RT22, IV—activation with CO_2_ (2 h) z RT22, V—activation with KOH with RT22.

**Figure 5:**
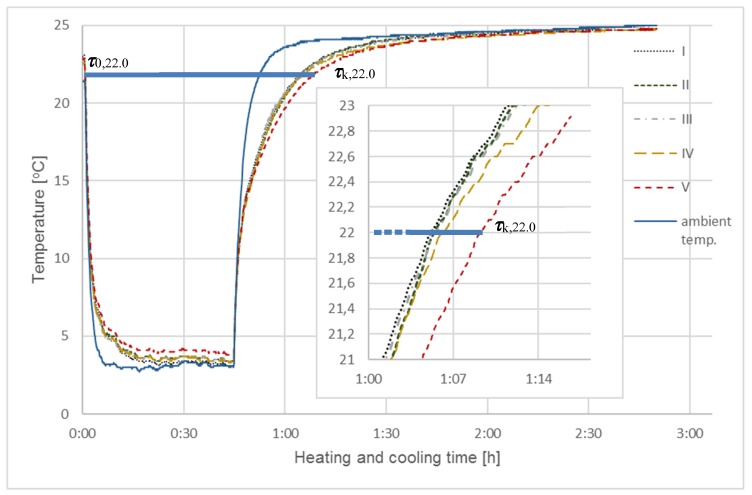
Results of natural heating for five chestnut char samples—SIII: I—without activation and without RT22, II—without activation with RT22, III—activation with CO_2_ (1 h) with RT22, IV—activation with CO_2_ (2 h) with RT22, V—activation with KOH with RT22.

Five different coatings were synthesized from the same chestnut char. Coatings corresponding to samples I and II contained non-activated char. Sample I was the reference sample, did not contain PCM (Rubitherm RT 22), and sample II did. The remaining samples all contained PCM; their char were activated, as follows: sample III—with CO_2_ (for 1 h), sample IV—with CO_2_ (for 2 h), and sample V—with KOH. The coating numbers correspond to the sample numbers in Figure 3, Figure 4 and Figure 5.

All of the coatings were conducted in the same manner. The same amount of char (0.2 g) with differing amounts, depending on the type of activation, of RT22 were ground in a mortar with one drop of saponin and seven drops of liquid PVA (poly(vinyl alcohol)) glue. After obtaining a homogeneous semi-liquid mass, it was applied to temperature sensors, placed in a stainless-steel cover (5 mm diameter, 30 mm long). 

After the coatings dried, the samples were attached between two slats (250 mm × 30 mm × 10 mm), according to the method that is shown in Figure 2, and subjected to two tests. The first procedure consisted of heating (0.5–1.0 h) samples with a solar simulator (infrared radiator in the form of a special reflector) in a thermostatic room, then cooling them (1.0 h) after switching off the radiator (Figure 3 and Figure 4). In the second procedure, the heating times and temperatures of samples previously cooled in the refrigerator were measured several times in a thermostatic room (Figure 5).

The infrared radiator that was used in these tests was located 0.5 m from the strip with the samples attached. The value of light intensity incident on the samples was *E*_f_ = 18.5 klux, and irradiation measured in the same place *E*_ir_ = 385 W/m^2^.

The measuring system consisted of eight DS18B20 temperature sensors. Five measured the coating temperatures; the other three measured temperatures in non-disturbed areas at distances between 1.5 and 2 m from the coatings. The temperature sensor cables were connected to the AVT 5330 module as well as the data processing and counting system. The obtained results were recorded on a computer that was equipped with the station.

Measurement uncertainties that were related to sensor accuracy were specified by the manufacturer as 0.25 °C. The standard deviation calculated for the sensors was 0.6 °C; the total measurement uncertainty was estimated as 0.85 °C.

The temperature of each sample was determined as a function of time, both during heating and cooling, based on these results. Figure 2 shows the overall diagram of the test stand.

## 4. Results of Thermodynamic Properties of the Obtained Material

In the first series of simulation tests (SI), all of the samples were exposed to infrared radiation for a period of 0.5 h. This made it possible to heat the sample coatings to a temperature approximating hot summer days (ambient temperature > 30 °C). After heating, the samples were allowed to cool to ambient temperature; the cooling times increased slightly during subsequent cycles (from ~19.7 °C (I cycle), 20.8 °C (II cycle) to 21.0 °C (III cycle)). The ambient temperature was taken as the average temperature from three sensors that were positioned at 1.5–2.0 m from the measuring station. The final temperature of the heated samples also increased during subsequent cycles and ranged from 28.5–30.0 °C in cycle I, 30.5–32 °C in cycle II, and 31.0–33.0 °C in cycle III. The heating and cooling tests in this and subsequent series were conducted in triplicate; Figure 3 presents the aggregate results that were obtained in the initial heating/cooling tests. The thick horizontal sections in the figure represent the three cycle times (Δ***τ*** = ***τ***_k,22.0_ − ***τ***_0,22.0_), measured for each of the samples upon exceeding 22.0 °C, (***τ***_0,22_), during heating until 22.0 °C was reached during cooling ***τ***_k,22_. Table 4 summarizes the cycle lengths obtained for this series (SI) and the next three (SII).

It was decided to compare the cycle times that were measured from ***τ***_0_, i.e., the moment when the sample exceeded the set initial temperature ***T***_0_ to the final time ***τ***_k_—i.e., the moment of the last reading of the set final temperature ***T***_k_. The temperatures were selected, so that the results of all measurement series could be compared, and their values were as close as possible to the phase transition temperature, which for Rubitherm RT22 is 22 °C.

In this study, instead of measuring the temperature increase Δ***T*** in given time frames (***τ***_0_, ***τ***_k_), the time intervals between the transition of sample temperatures from ***T***_0_ to ***T***_k_ were measured, due to greater accuracy of time measurement than the accuracy of temperature increase measurement, especially in fast-changing processes.

This procedure was repeated for a second series of tests (SII) with an increase in the sample heating time to 1 h (Figure 4). This resulted in a slight final temperature increase that was nearly identical to the results that were obtained for heating for 30 min. The final ambient temperatures at the end of each cycle in both procedures were practically the same; however, fluctuations in ambient temperatures during individual cycles in the second series were greater. This slightly influenced the maximum coating temperature level, but proved that a longer exposure time in this case is not intentional and the assumed cooling time of one hour was sufficient for describing the cooling phenomenon; after that one hour, all of the samples reached the same temperature and they also cooled at the same rate.

Both of the studies on the heating and cooling process of char containing RT22 confirmed that there was a clear difference between temperature changes as a function of time obtained for the individual samples. Differences in the cooling rates were more pronounced than the heating rates. Radiative heat exchange during heating was more intense than during convection cooling, which steepens the heating curves of individual samples and differences in the heat exchange time between individual samples become less visible. The coatings were thin, as were the amounts and mass of char, and Rubitherm RT22 contained in them in relation to the weight of the temperature sensors. However, the effect of the absorbed RT22 on the heat capacity of the modified char turned out to be sufficiently clear, especially on the cooling curves. It was concluded that, for larger amounts of PCM modified carbonate, in relation to the mass of the sensor, this effect will be even greater due to the change of heat accumulation from the surface to the interior.

The comparative analyses shown in Figure 3 and Figure 4 indicated that extended sample heating time did not impact the cooling curve shape, which were almost identical for every cycle in the first two testing series. However, longer heating times did cause greater dissection and flattening of the heating curves for individual samples, especially after exceeding the phase transition temperature of Rubitherm RT22 (22 °C, according to manufacturer specifications and the results in Table 3). However, after heating the thin char coating, additional heating was carried using a temperature sensor with a much higher mass and heat capacity than the tested coatings.

Differences in individual sample final temperatures, which are visible in all test cycles carried out in both series, come from the termination of the sample heating process after exceeding the phase transition temperature of Rubitherm RT22, but before they reach thermodynamic equilibrium.

A most interesting aspect of this research corresponds to temperatures near the phase transition temperature, at which the storage and release of thermal energy occurs. This was observed and measured for both series, though the impact of Rubitherm RT22 was only sufficiently clear for sample cooling. The excessively high sample heating rate masked the effects of differences that are caused by phase change in Rubitherm. As such, a third series of tests (SIII) was devised, during which the heating rate was reduced and the final temperature was lowered to better correspond to the RT22 phase transition temperature.

The SIII series was carried out using only one cooling and heating cycle, as the results that were obtained in the previous SI and SII series showed that repeating the cycles resulted in no changes. For this series, samples were initially cooled to 3 °C for 30 min., placed in a room maintained at 25 °C, and were then heated automatically and at a much slower rate than radiant heating. Those results, together with the enlarged fragment, enabled a more accurate analysis of the heating curves and a more qualitative assessment of the differences that were obtained for individual samples; those are presented in Figure 5.

As expected, the heating curve that was obtained in this experiment was more inclined, which made it easier to analyze differences in the sample process times.

The results that were obtained for SI and SII for all three test cycles, determining the total time of full cycles measured from the initial temperature ***T***_0_ = 22 °C, by heating–cooling, until the individual samples reach the final temperature ***T***_k_ = 22 °C, are summarized in Table 4. Table 4 also shows the results obtained in SIII over the same temperature range, though that cycle involved cooling the samples prior to heating them. A graphical illustration of the results that are listed in Table 4 are thick horizontal sections in Figure 3, Figure 4 and Figure 5, determining the cumulative times for full heating and cooling cycles or cooling/heating for all samples.

## 5. Analysis and Discussion

Analyzing curves in Figure 3, Figure 4 and Figure 5, and combined with results collected in Table 4, selected at one temperature (22 °C), clearly indicated the impact of different Rubitherm RT22 amounts in the char on the cycle time length Δ***τ*** = ***τ***_k,22.0_ − ***τ***_0,22.0_ of heat exchange processes taking place in individual samples. Differences in the process times are visible, even though the amount of Rubitherm RT22 adsorbed was small relative to the much higher mass and thermal capacity of the temperature sensors.

When compared to the reference sample (I) with no Rubitherm RT22, the total cooling and heating times (Δ***τ*** = ***τ***_0,22.0_ − ***τ***_k,22.0_) of all samples with differing levels of Rubitherm RT22 were longer. For sample V (activated with KOH), this time was extended by 7.5%, 5.2% for sample IV, 2.4% for III, and only 0.2% for sample II (non-activated char).

It was decided to reanalyze the obtained results to better understand the effect of char with different contents of Rubitherm RT22 on the mechanism of heat exchange occurring in the samples. It was conducted by eliminating fast-changing heat exchange processes and only focusing on the cooling times of the samples in the SI and SII series (Table 5), and their heating times in SIII (Table 6).

The results obtained in SI and SII included in Table 5 were selected because they featured the widest temperature range of all cycles (between 21.5–28.5 °C). The results obtained in SIII, because they cover a different temperature range (5.0–23.5 °C) and in a different heating/cooling order, are separately presented in Table 6.

The results in Table 4, Table 5 and Table 6 indicated that Rubitherm RT22 contained in char affected heat exchange during the heating and cooling of the samples. However, during cooling, as the RT22 content in char increased, the sample cooling time also increased relative to the reference sample (I), which did not contain Rubitherm. This is clearly seen in the last row of Table 5, which clearly shows the average cooling time extensions for all SI and SII cycles, which are: 26.4% for sample V, 14.8% for sample IV, 11.7% for sample III, and 3.6% for sample II, relative to sample I. The cooling time differences of individual samples are greater than differences in full cycle times (Table 4). This is due to the much larger heat capacity of the temperature sensor relative to the mass of the RT22 char tested. The extension of the total heating and cooling times caused by this resulted in a reduction of the differences in the individual samples. The results collected in Table 5 were analyzed at shorter cooling times, limited by a lower temperature range (28.5–21.5 °C). This elimination of the effect on the heating time results allowed for an observation of larger differences in cooling times between individual samples.

Fearing that the rapid heating rate of the samples in SI and SII might affect result interpretations, which may be ambiguous, accidental, and contradictory, a modified SIII experiment was planned and carried out.

The order of heating and cooling processes was changed with the rate and final heating temperature lowered. Consequently, it was possible to obtain less steep heating curves (Figure 5), from which it was easier to observe heating time differences of individual samples. 

At high rates of temperature change, e.g. steep heating curves in Figure 3 and Figure 4, as well as cooling curves in Figure 5, fast temperature changes take place in a short period. As time was measured in these studies, the shorter it was, the less accurate the results were. On less steep cooling (Figure 3 and Figure 4) or heating (Figure 5) curves, differences in the time to reach the same temperature could already be measured with much greater accuracy that allowed for the differences between individual samples to be determined.

The results obtained in SIII, as summarized in Table 6 for heating time, show a similar, but inverse, trend to those in Table 5; the effect of Rubitherm RT22 on changes in heat transfer times for individual samples. This time, cooling curves obtained in SIII—opposite to those in SI and SII—were too steep to determine the effect of Rubitherm RT22 levels; therefore, they were excluded from consideration. Table 6, instead, focuses on results that are related to heating times.

The interpretation of the heating process time results was unambiguous and consistent with the results obtained in SI and SII, which are presented in the bottom row of Table 5. As the levels of Rubitherm RT22 in the char increased, so did the sample heating times: by 22.1% (V), 16.0% (IV), and 4.1% (III). Only for sample II was there a slight, within the margin of error (0.5%) heating time decrease relative to the reference sample (I).

These results obtained for SI and SII and collected in Table 5, as well as the results obtained in SIII for the heating process (Table 6), are unambiguous and clearly indicate that Rubitherm RT22 contained in the carbonate directly impacts the thermal processes occurring in it. During phase transitions (melting-solidification), thermal energy is stored and then released; this work clearly documents the extension of heat transfer times.

## 6. Conclusions

A novelty in this work is the research and testing of a new carrier for phase-change material. Thus far, no one has tried to utilize pyrolytic biochar with a well-developed internal surface for permanent PCM adsorption (due to the van der Waals forces).

The proposed and conducted research procedure of cooling during SI and SII series and heating in SIII, designed for small masses of tested char RT22 carrier (about 0.2 g) and small thicknesses of coatings (0.1–0.15 mm), proved to be so accurate that it provided unambiguous results that were consistent with data obtained at the remaining stages of the study. These measurements, as well as the measurements of the degree of activation for various methods of activated carbon obtaining, and the adsorption studies showed that the amount of Rubiterm RT22 is the highest in the activated KOH carbonate (sample V), smaller in activated 2 h CO_2_ (sample IV), even smaller in activated 1 h CO_2_ (sample III), and the smallest in non-activated carbonate (sample II).

The results showed that the use of char as a PCM carrier meets expectations regarding accumulation and slower heat release to the environment. Heating and cooling tests both showed similar dynamics of increases/decreases during heat exchange and represent a function of its accumulation and release during phase transitions.

Thermal analysis tests using DSC confirmed the potential of using carbonate as a potential PCM carrier. Further simulation studies, consisting of measuring heating/cooling times of coatings made of RT22 modified char, further confirmed that properly activated char that was obtained from waste biomass had similar thermodynamic parameters to commercial materials of this type upon the absorption of PCM, but were much less expensive than commercial products.

In addition, it has been shown that PCM impregnated carbonate materials have significant pro-ecological values because:

- it is a recycled product of waste biomass subjected to torification or pyrolysis;
- char as a PCM carrier is an inexpensive, chemically, and environmentally neutral product; and,
- the PCM used in these studies is a hydrocarbon derived from the distillation of crude oil, a type of paraffin that is chemically unreactive and, therefore, environmentally neutral.

Thanks to its well-developed surface and high absorption capacity, the activated char permanently adsorbs such an amount of PCM that obtaining up to 50% greater heat capacity in relation to RT22 is possible and, due to van der Waals forces, the PCM will not be desorbed, even in liquid state.

The use of the char modified in the above manner, in the form of plaster addition, paints, screeds, asphalt mixtures, etc., in addition to stabilizing the temperature, storing temporary excess energy, and releasing it in a timely fashion, also prevents local overheating, both on the surface and within an object. Overheating can lead to softening, flowing, thermal deformation and degradation by oxidation, drying, or cracking; all of which can lead to material damage as well as objects that are made of them.

Research conducted in this work proves that the development of such a building material, which, when compared to commercial products, will be much cheaper, equally effective, and possible to use in the production of gypsum boards, plasters, screeds, etc., just as Micronal, is only a matter of time. However, detailed research on the implementation of this new material will be the subject of a separate paper.

The continuation of this research topic is justified because the research showed that biochar can be a PCM carrier, and even a coating created from it clearly affects the time of heat transfer. Heat transfer studies should be conducted—no longer on the surficial scale (2D), however, but on the capacitively-volumetric (3D) scale. Larger amounts of PCM modified biochar will be required to carry out this research and, thus, the scale of research conducted in this work (pyrolysis, activation, sorption) should be increased from a laboratory scale to a technical or semi-technical one.

## Figures and Tables

**Table 1:** Raw waste material properties.

	Proximate Analysis (wt.%)			Elemental Analysis (wt.%)				
Sample	Moisture	Ash	Volatiles	C	H	N	O	S
***Chestnut***	3.5	3.5	74.6	43.6	6.6	1.4	47.8	0.2
***Chestnut char***	-	-	-	74.3	1.1	1.8	24.3	0.0

**Table 2:** Surface area (S_BET_) and pore size (V_p_) of activated carbon obtained from the chestnut.

	Material	Chestnut	
No	Activation Method	S_BET_ [m^2^/g]	V_p_ [cm^3^/g]
**I, II**	Reference Samples	31.6	0.016
**III**	CO_2_ (1 h)	58.7	0.030
**IV**	CO_2_ (2 h)	143.3	0.073
**V**	KOH	1252.5	0.643

**Table 3:** Comparison of DSC results for different char samples, including; carbonate from chestnut fruit: A—not activated, B—activated with CO_2_ (1 h), C—activated with CO_2_ (2 h), D—activated with KOH; char from: E—coconut shell activated with CO_2_ (1/2 h), F—walnut shell activated with CO_2_ (1/2 h), and reference samples: G—pure Rubitherm RT22 and H—Micronal DS 5040 X.

	A	B	C	D	E	F	G	H
**Sample Weight [mg]**	5.27	5.48	5.78	5.59	5.51	6.16	6.52	6.45
**Enthalpy *ΔH* [kJ/kg]**	49.2	67.1	84.0	125.7	42.9	66.2	256.4	150.4
Onset Temp. *T* * [°C]	25.3/24.6	23.0/22.9	23.4/23.7	22.8/22.3	23.3/22.9	23.2/22.3	21.7/21.6	18.6/17.7
Peak Temp. *T* ** [°C]	28.0/23.6	26.2/21.1	26.2/21.4	25.6/21.3	25.3/22.0	26.1/21.8	26.9/17.8	25.5/15.3

(*) measured at phase change onset for heating/cooling process (**) heating/cooling process.

**Table 4:** Summary of individual heating and cooling cycle durations for coatings made from the tested char with different Rubiterm RT22 content.

Samples	I	II	III	IV	V
**SI—First Heating-Cooling Cycle**					
***τ***_k,22,0_	00:39:44	00:39:44	00:39:44	00:39:44	00:39:44
***τ***_0,22.0_	00:02:10	00:02:32	00:02:14	00:02:04	00:02:16
Δ***τ*** = ***τ***_k,22.0_ − ***τ***_0,22.0_	00:37:34	00:37:12	00:37:30	00:37:40	00:37:28
Percentage	100	99.7	103.1	106.6	108.6
**SI—Second Heating-Cooling Cycle**					
***τ***_k,22,0_	02:14:10	02:14:16	02:15:14	02:16:54	02:18:26
***τ***_0,22.0_	01:29:10	01:29:20	01:29:12	01:29:06	01:29:10
Δ***τ*** = ***τ***_k,22.0_ − ***τ***_0,22.0_	00:45:00	00:44:56	00:46:02	00:47:48	00:49:16
Percentage	100	99.8	102.3	106.2	109.5
**SI—Third Heating-Cooling Cycle**					
***τ***_k,22,0_	03:51:11	03:51:11	03:53:05	03:55:31	03:57:57
***τ***_0,22.0_	02:58:21	02:58:25	02:58:21	02:58:19	02:58:21
Δ***τ*** = ***τ***_k,22.0_ − ***τ***_0,22.0_	00:52:50	00:52:46	00:54:44	00:57:12	00:59:36
Percentage	100	99.9	103.6	108.3	112.8
**SII—First Heating-Cooling Cycle**					
***τ***_k,22,0_	01:15:38	01:15:26	01:16:18	01:17:30	01:18:32
***τ***_0,22.0_	00:02:28	00:02:46	00:02:30	00:02:18	00:02:32
Δ***τ*** = ***τ***_k,22.0_ − ***τ***_0,22.0_	01:13:10	01:12:40	01:13:48	01:15:12	01:16:00
Percentage	100	99.3	100.9	102.9	103.9
**SII—Second Heating-Cooling Cycle**					
***τ***_k,22,0_	03:26:29	03:26:41	03:28:05	03:30:07	03:31:57
***τ***_0,22.0_	02:01:21	02:01:29	02:01:21	02:01:17	02:01:21
Δ***τ*** = ***τ***_k,22.0_ − ***τ***_0,22.0_	01:25:08	01:25:12	01:26:44	01:28:50	01:30:36
Percentage	100	100.1	101.9	104.3	106.4
**SII—Third Heating-Cooling Cycle**					
***τ***_k,22,0_	05:36:53	05:38:07	05:40:05	05:40:59	05:43:23
***τ***_0,22.0_	04:01:01	04:01:05	04:01:01	04:01:01	04:01:01
Δ***τ*** = ***τ***_k,22.0_ − ***τ***_0,22.0_	01:35:52	01:37:02	01:39:04	01:39:58	01:42:22
Percentage	100	101.2	103.3	104.8	106.8
**SIII—Heating-Cooling Cycle**					
***τ***_k,22,0_	01:05:02	01:05:18	01:05:14	01:05:54	01:09:10
***τ***_0,22.0_	00:00:42	00:00:42	00:00:42	00:00:42	00:00:42
Δ***τ*** = ***τ***_k,22.0_ − ***τ***_0,22.0_	01:04:20	01:04:36	01:04:32	01:05:12	01:08:28
Percentage	100	100.4	100.3	101.3	106.4
**Average**	**100**	**100.2**	**102.4**	**105.2**	**107.5**

**Table 5:** Summary of cooling times of coatings from temperature ***T***_0_ = 28.5 °C to temperature ***T***_k_ = 21.5 °C for series SI and SII.

Samples	I	II	III	IV	V
**SI—First Cooling Cycle**					
***τ***_0,28.5_	00:29:58	00:29:44	00:30:02	00:30:22	00:30:06
***τ***_k,21.5_	00:42:36	00:43:20	00:44:58	00:46:20	00:47:44
Δ***τ*** = ***τ***_k,21.5_ − ***τ***_0,28.5_	00:12:38	00:13:36	00:14:56	00:15:58	00:17:38
Percentage	100	107.6	118.2	126.4	139.6
**SI—Second Cooling Cycle**					
***τ***_0,28.5_	01:58:48	01:58:40	01:58:58	01:59:22	01:59:06
***τ***_k,21.5_	02:19:38	02:20:08	02:21:40	02:22:54	02:26:04
Δ***τ*** = ***τ***_k,21.5_ − ***τ***_0,28.5_	00:20:50	00:21:28	00:22:42	00:23:32	00:26:58
Percentage	100	103.0	100.0	113.0	129.4
**SI—Third Cooling Cycle**					
***τ***_0,28.5_	03:29:27	03:29:13	03:29:35	03:29:59	03:29:47
***τ***_k,21.5_	04:03:27	04:04:33	04:09:15	04:09:09	04:13:37
Δ***τ*** = ***τ***_k,21.5_ − ***τ***_0,28.5_	00:34:00	00:35:20	00:39:40	00:39:10	00:43:50
Percentage	100	103.9	116.7	115.2	128.9
**SII—First Cooling Cycle**					
***τ***_0,28.5_	01:01:18	01:01:08	01:01:26	01:01:48	01:01:32
***τ***_k,21.5_	01:19:00	01:18:56	01:20:04	01:22:12	01:23:36
Δ***τ*** = ***τ***_k,21.5_ − ***τ***_0,28.5_	00:17:42	00:17:48	00:18:38	00:20:24	00:22:04
Percentage	100	100.6	105.3	115.2	124.7
**SII—Second Cooling Cycle**					
***τ***_0,28.5_	03:02:47	03:02:31	03:02:55	03:03:21	03:03:15
***τ***_k,21.5_	03:36:47	03:37:55	03:40:51	03:41:23	03:45:19
Δ***τ*** = ***τ***_k,21.5_ − ***τ***_0,28.5_	00:34:00	00:35:24	00:37:56	00:38:02	00:42:04
Percentage	100	104.1	111.6	111.9	123.7
**SII—Third Cooling Cycle**					
***τ***_0,28.5_	05:03:35	05:03:19	05:03:41	05:04:09	05:04:07
***τ***_k,21.5_	05:54:12	05:55:04	05:59:16	05:58:16	06:00:58
Δ***τ*** = ***τ***_k,21.5_ − ***τ***_0,28.5_	00:50:37	00:51:45	00:55:35	00:54:07	00:56:51
Percentage	100	102.2	109.8	106.9	112.3
**Average**	**100**	**103.6**	**111.7**	**114.8**	**126.4**

**Table 6:** Summary of coating heating times during the third round of measurements from ***T***_0_ = 5.0 °C to ***T***_k_ = 23.5 °C and their cooling from ***T***_0_ = 23.5 °C to ***T***_k_ = 5.0 °C for SIII.

Samples	I	II	III	IV	V
**SIII—heating round**					
***τ***_0,5.0_	00:45:12	00:45:10	00:45:12	00:45:12	00:45:10
***τ***_k,23.5_	01:18:12	01:18:00	01:19:34	01:23:28	01:25:28
Δ***τ*** = ***τ***_k,23.5_ − ***τ***_0,5.0_	00:33:00	00:32:50	00:34:22	00:38:16	00:40:18
Percentage	**100**	**99.5**	**104.1**	**116.0**	**122.1**
